# Neurotoxicity Caused by the Treatment with Platinum Analogues

**DOI:** 10.1155/2011/843019

**Published:** 2011-06-27

**Authors:** Sousana Amptoulach, Nicolas Tsavaris

**Affiliations:** Oncology Unit, Department of Pathophysiology, Laikon General Hospital, Athens University School of Medicine, 11527 Athens, Greece

## Abstract

Platinum agents (cisplatin, carboplatin, and oxaliplatin) are a class of chemotherapy agents that have a broad spectrum of activity against several solid tumors. Toxicity to the peripheral nervous system is the major dose-limiting toxicity of at least some of the platinum drugs of clinical interest. Among the platinum compounds in clinical use, cisplatin is the most neurotoxic, inducing mainly sensory neuropathy of the upper and lower extremities. Carboplatin is generally considered to be less neurotoxic than cisplatin, but it is associated with a higher risk of neurological dysfunction if administered at high dose or in combination with agents considered to be neurotoxic. Oxaliplatin induces two types of peripheral neuropathy, acute and chronic. The incidence of oxaliplatin-induced neuropathy is related to various risk factors such as treatment schedule, cumulative dose, and time of infusion. To date, several neuroprotective agents including thiol compounds, vitamin E, various anticonvulsants, calcium-magnesium infusions, and other nonpharmacological strategies have been tested for their ability to prevent platinum-induced neurotoxicity with controversial results. Further studies on the prevention and treatment of neurotoxicity of platinum analogues are warranted.

## 1. Introduction

Platinum drugs are among the most important cytotoxic drugs available to oncologists. Although they share some structural similarities, there are also marked differences in their therapeutic use, pharmacokinetics, and adverse effect profiles [1–3]. Cisplatin is the first agent of platinum drugs, which was approved in 1978 for the treatment of testicular and ovarian cancer [4]. In view of its considerable toxicity profile, many attempts have been made to develop analogues with less toxicity, increased efficacy, or both. Carboplatin is a second-generation platinum drug with equivalent activity, in some cancer types, to cisplatin. Carboplatin is often administered in combination with a taxane as a first-line treatment for ovarian cancer [5, 6]. Lung cancer is also treated with carboplatin in combination with vinorelbine, gemcitabine, or paclitaxel [7]. Oxaliplatin, a third-generation platinum drug, is the standard of treatment, together with 5-fluorouracil/leucovorin (5 FU/LV), for locally advanced and metastatic cancer of the colon and rectum [8]. This paper aims to highlight the neurotoxicity of commonly used platinum agents and published data on certain compounds that have been reported to have protective effects when administered simultaneously with these agents. 

## 2. Cisplatin

Peripheral neurotoxicity is the most important dose-limiting problem associated with cisplatin [9]. A number of pathophysiological mechanisms have been proposed to explain this phenomenon, with some data suggesting that cisplatin kills malignant cells and peripheral neurons by means of a similar mechanism of apoptosis [10]. Peripheral neurotoxicity develops in approximately 50% of patients receiving cisplatin [11], but the onset of toxicity is delayed until a cumulative dose higher than 300 mg/m^2^ has been given [12, 13]. Signs and symptoms of peripheral neurotoxicity involve the upper and lower extremities and include loss of vibration sense, loss of position sense, tingling paraesthesia, weakness, tremor, and loss of taste [14–16]. Seizures and leukoencephalopathy have also been described [17, 18]. After discontinuation of treatment, the neurological dysfunction may gradually improve, but it may persist for a period of time, or it can be permanent [11, 19].

Cisplatin is ototoxic. Tinnitus and hearing loss have been observed in up to 31% of patients treated with initial intravenous cisplatin dose of 50 mg/m^2^ [20, 21]. Transient hearing loss and mild audiometric abnormalities were observed in 30% of patients receiving 150 mg/m^2^ of cisplatin [22, 23]. The mechanisms of cisplatin-induced damage to the outer hairy cells of the cochlea probably include the formation of reactive oxygen radicals and depletion of glutathione [24]. Other risk factors include simultaneous use of other potentially ototoxic agents (e.g., aminoglycosides), previous cranial irradiation, preexisting renal dysfunction, or inner ear damage [21, 23, 25, 26]. 

## 3. Carboplatin

Carboplatin is considered to be less neurotoxic than cisplatin [27]. Neurological dysfunction is a side effect in the carboplatin-based regimen but appears later on and mostly after the administration of carboplatin at high-dose levels or in combination with other cytotoxic agents known to be neurotoxic (e.g., taxanes) [5, 6, 28]. Only 4–6% of patients who receive carboplatin may develop peripheral neuropathy [29]. Pretreated patients with other neurotoxic agents (e.g., cisplatin, and etoposide) and those who are older than 65 years have a higher risk [29, 30].

Ototoxicity, after therapy with carboplatin, is thought to be rare. A small proportion of patients, about 1.1%, show symptoms such as tinnitus or subclinical audiographical changes [31]. 

## 4. Oxaliplatin

Oxaliplatin is more like cisplatin in its potential to produce significant neurological dysfunction (Table 1). Peripheral neuropathy is the most common dose-limiting toxicity of oxaliplatin, and it is one of the major causes of discontinuation of therapy. Sensory peripheral neuropathy caused by administration of oxaliplatin is distinguished in 2 forms: (1) an acute peripheral sensory neuropathy that may appear during the administration of the drug or after the first few drug infusions and (2) a chronic dose-limiting cumulative peripheral sensory neuropathy. The mechanisms underlying these two forms of oxaliplatin-induced peripheral neuropathy have not been clearly defined. Acute neurotoxic effects may result from the impairment of voltage-gated sodium channels and occur approximately in 85–95% of all patients exposed to oxaliplatin [32, 33]. Symptoms consist mainly of paresthesias and dysesthesias in the extremities and the perioral region and are exacerbated by cold exposure.

One other very rare manifestation of acute oxaliplatin-induced neurotoxicity is laryngopharyngeal dysesthesia, a transient sensation of difficulty in breathing without evidence of respiratory distress [20, 34]. This transient syndrome affects approximately 1–2% of patients and recovers between cycles [35]. The risk of acute neuropathy appears to be lower if oxaliplatin is administrated in a dose of 85 mg/m^2^ every 2 weeks rather than 130 mg/m^2^ every 3 weeks [33, 34]. 

The most accepted mechanism of the chronic form oxaliplatin-induced neurotoxicity is decreased cellular metabolism and axonplasmatic transport resulting from the accumulation of oxaliplatin in the dorsal root ganglia cells. As a result, oxaliplatin produces symmetrical, axonal, and sensory distal neuropathy [36]. Neurological symptoms in this form of sensory neurotoxicity are dominated by pronounced paresthesias and dysesthesias of the extremities and dysfunction of fine sensory-motor coordination which may result in impairment of daily life [20, 37]. Other rare atypical neurosensory symptoms associated with higher cumulative dose of oxaliplatin (higher than 1000 mg) are those of spinal cord compression (Lhermitte sign) and urinary retention [38]. The incidence of chronic oxaliplatin-induced peripheral neuropathy is related to various risk factors such as cumulative dose, treatment schedule, and time of perfusion [39]. The peripheral sensory neuropathy induced by oxaliplatin tends to improve after treatment is stopped. Symptoms are partly reversible in approximately 80% of patients and resolve completely in about 40% of patients 6–8 months after the discontinuation of oxaliplatin treatment [36, 40].

Ototoxicity due to administration of oxaliplatin is very uncommon [41, 42]. 

## 5. Prevention and Treatment of Platinum-Induced Neurotoxicity

Many studies have examined the efficacy of a number of potential neuroprotective agents administered together with platinum analogues. The use of these agents generally aims to reduce the incidence and severity of the neurotoxicity without impairing the antitumor efficacy of the platinum drugs. 

### 5.1. Thiol Compounds

Three thiol compounds have been studied as neuroprotective agents in patients receiving cisplatin: amifostine, glutathione, and the melanocortin Org 2766. 

Among these agents, glutathione seems to have some neuroprotective effects in platinum-induced neurotoxicity. Glutathione has been studied as a chemoprotective agent in patients receiving chemotherapy with cisplatin in small randomized trials. Published data are conflicting as some of the studies have shown that glutathione may provide neuroprotection in patients treated with cisplatin without altering its antineoplastic effect [37, 43, 44], while others found no reduction in toxicity [45, 46]. Cascinu et al. were the first to study the potentially protective effect of glutathione on oxaliplatin neurotoxicity in a randomized, placebo-controlled clinical trial [47]. This study showed that glutathione can exert a beneficial effect on oxaliplatin-induced neurotoxicity without interferences with oxaliplatin antitumor activity. Other studies, however, have shown no benefit from the use of glutathione for preventing the oxaliplatin-induced peripheral neuropathy [48]. In addition, the fact that elevated intracellular levels of glutathione have been correlated with increased resistance to platinum agents raises some concerns over the use of glutathione as a neuroprotective agent [48–50]. More trials are needed to confirm the safety and usefulness of glutathione as a protective agent against platinum-induced neurotoxicity.

In respect to cisplatin-induced ototoxicity, there is some evidence that amifostine may be useful as a protective agent [20, 51]. 

### 5.2. Vitamin E

The neuroprotective role of vitamin E against cisplatin neurotoxicity has recently been evaluated by Pace et al. in a randomized, placebo-controlled trial [52]. This was a phase III study in which 108 patients, treated with cisplatin, were randomized to receive vitamin E (alpha-tocopherol 400 mg/day) or placebo. Class II evidence that vitamin E supplementation significantly reduces the relative risk of developing signs or symptoms of neurotoxicity (relative risk = 0.14) (95% confidence interval = 0.02–1.00, *P* < 0.05) was provided [52]. 

### 5.3. Calcium (Ca) and Magnesium (Mg) Infusions

Ca/Mg infusions have been used to decrease the incidence of oxaliplatin-induced neuropathy without any influence on antitumor activity [53–55]. However, the CONCEPT study reported that treatment with Ca/Mg decrease antitumor effect in patients with metastatic colorectal cancer treated with oxaliplatin, and thus, they are not advisable in combination with the FOLFOX regimen [56]. 

### 5.4. Anticonvulsants (Gabapentin and Pregabalin)

Gabapentin is an antiepileptic drug which has been used in the management of neuropathic pain. Some studies attempted to assess the impact of gabapentin on oxaliplatin-induced neurotoxicity, but they could not support a role for gabapentin in reducing the severity of oxaliplatin-induced neurotoxicity [57–59]. 

Pregalin is also an anticonvulsant drug used for neuropathic pain. A case of successful treatment of hyperexcitability syndrome with pregalin after oxaliplatin and gemcitabine therapy for pancreatic cancer has been described [60]. In a recent study, Saif et al. treated 23 patients with gastrointestinal tumors and grade 2 and 3 oxaliplatin-induced neurotoxicity with pregabalin at a dose of 150 mg orally 3 times a day and found that pregabalin reduced the severity of oxaliplatin-induced neuropathy [61]. 

### 5.5. Other Strategies

Nonpharmacological approaches to prevent oxaliplatin-induced neurotoxicity include the “stop and go” concept. This strategy is based on the observation of reversibility of neurotoxic symptoms after discontinuation of oxaliplatin. In this approach, patients receive treatment with oxaliplatin plus 5 FU/LV until either the beginning of the development of peripheral neurotoxicity or a predetermined “time to best response”. Next, maintain ace therapy with 5 FU/LV is continued without oxaliplatin so that any neurologic damage is given time to recede. Subsequently, oxaliplatin is reintroduced to maximize the potential effect of the combination regime [62].

Another strategy suggested by Petrioli et al. involves longer duration of oxaliplatin administration which is supposed to result in decreased neurotoxicity [63]. 

## 6. Conclusions

Platinum compounds are active in the treatment of solid tumors, but peripheral neuropathy is the major non-hematological dose-limiting adverse effect especially for cisplatin and oxaliplatin. Despite the efforts to find specific agents who will prevent or minimize neurotoxicity caused by platinum drugs, there is no effective strategy for the management of the neurotoxicity induced by platinum agents. Unfortunately, none of the symptomatic treatments discussed above have proven useful. Therefore, new drugs or strategies for the prevention and amelioration of platinum-induced neurotoxicity must be found. 

## Figures and Tables

**Table 1:** 

Comparison of cisplatin- and oxaliplatin-induced neurotoxicity	Cisplatin	Oxaliplatin
Dose limiting toxicity	Peripheral neurotoxicity	Peripheral neurotoxicity
Symptoms	Paresthesia	Paresthesia, sensory ataxia, and dysesthesia
Location	Extremities	Extremities, perioral area
Time-course onset	Delayed	Acute and delayed
After treatment	deterioration	Recovery
Accompanying toxicities	Ototoxicity	Laryngospasms
Precipitating factors	None	Exposure to cold
